# Differences found in patient characteristics of migrant tuberculosis sub-populations within low TB incidence European countries, 2014–2020

**DOI:** 10.1186/s12879-025-11085-0

**Published:** 2025-06-02

**Authors:** Sarah Jackson, Barbara Hauer, Jean-Paul Guthmann, Mary O´Meara, Vinciane Sizaire, Karine Nordstrand, Anders Koch, Brit Häcker, Gerard de Vries, Jerker Jonsson, Kristina Langholz Kristensen, Wouter Arrazola de Oñate, Hanna Soini, Teresa Domaszewska

**Affiliations:** 1 Health Protection Surveillance Centre, Health Service Executive, Dublin, Ireland; 2 Department of Infectious Disease Epidemiology, Robert Koch Institute, Berlin, Germany; 3 Santé Publique France, Saint-Maurice, Paris, France; 4 Health Service Executive, National Health Protection Office, Dublin, Ireland; 5 FARES, Brussels, Belgium; 6 Norwegian Institute of Public Health, Oslo, Norway; 7 Statens Serum Institut, Copenhagen, Denmark; 8 Rigshospitalet University Hospital, Copenhagen, Denmark; 9 German Central Committee Against Tuberculosis (DZK), Berlin, Germany; 10 National Institute for Public Health and the Environment (RIVM), Bilthoven, Netherlands; 11 Folkhälsomyndigheten/Public Health Agency of Sweden, Stockholm, Sweden; 12 Department of Pulmonary and Infectious Diseases, Nordsjaellands Hospital, Hilleroed, Denmark; 13 BELTA - Belgian Lung and Tuberculosis Association, Brussels, Belgium; 14 Finnish Institute for Health and Welfare, Helsinki, Finland

**Keywords:** Tuberculosis, Epidemiology, Incidence, Public health, Emigration and immigration

## Abstract

**Introduction:**

In low TB incidence countries, prevention and care activities addressing migrant populations are essential for TB control. Understanding characteristics of TB patients in the migrant population is important for planning and providing appropriate care. This study aims to inform prevention and care strategies by describing characteristics of TB patients within migrant subpopulations in Europe to understand whether differences exist in their patient profiles.

**Methods:**

This cross-sectional descriptive study of migrants with TB reported to the European Surveillance System (2014–2020) from 23 low incidence European countries describes characteristics of different subgroups, according to TB epidemiological indicators and interval between arrival and notification.

**Results:**

Migrants with TB originating from very high TB incidence countries had the highest proportion of people living with HIV (7%) and highest extrapulmonary TB proportion (44%). Patients from high incidence countries had the highest proportion with previous TB diagnosis (14%), first line (12%) and multidrug (6%) resistances. Compared to all patients, patients arriving from the 10 countries with the highest crude incidence rates were on average 9 years younger (median age 25 vs 34) and more often male (M:F ratio 2.6 vs. 1.8). Patients notified < 2 years after arrival had higher proportions diagnosed with PTB (67%) and MDR-TB (4%), as well as people living with HIV (7%).

**Discussion:**

Unique patterns in patient characteristics were observed which varied by origin and destination. Improving European TB preparedness within the context of migration requires timely and complete international data alongside continuous access to quality TB care, not only at entry, and expanded opportunities for diagnosis given levels of extrapulmonary TB observed.

**Supplementary Information:**

The online version contains supplementary material available at 10.1186/s12879-025-11085-0.

## Background

In many low tuberculosis (TB) incidence countries (< 10 per 100,000 population), a large proportion of TB patients are migrants [1]. While the incidence in the non-migrant population has declined in most countries of the European Union (EU) and European Economic Area (EEA), the proportion of migrants among TB patients has increased, from 27% in 2014 to 33% in 2021. Higher proportions are observed in northern and western European countries [2]. Particularly in 2015–2016, an increase in global migration resulted in an increase of migrant TB patients [3]. In 2020, nearly 87 million international migrants lived in Europe, which is a further increase of nearly 16% since 2015 [4].

Consequently, TB prevention and care activities addressing migrant populations are an increasingly important part of TB programmes [5]. Policies vary among EU/EEA member states, but most often focus on active case finding, including screening at entry and contact tracing [5]. To plan and provide efficient health care strategies, understanding the differences between patient characteristics of various migrant subpopulations is crucial. The risk of acquiring and developing TB and consequently the impact on TB epidemiology in the destination countries is influenced by where people migrate from, why and how they travel, and how long ago they arrived [6]. Indeed, the individual TB risk depends on the intensity of TB transmission in the country of origin but also on the conditions related to migration, such as spending long periods in overcrowded camps, poor nutrition during migration, the stress generated by crossing dangerous borders or the fear of being sent back home by the authorities of the countries which they migrate through or to [1]. The risk of TB may also be influenced by the effectiveness of the screening strategy upon arrival in the destination country, living conditions after resettlement and access to the healthcare system [7, 8]. While some of these factors cannot be evaluated based on European level surveillance data, as respective variables are not available or collected in a harmonized way (e.g. information on migration route), we can characterise TB patients in migrant subpopulations based on key variables such as origin, destination, and interval between arrival and diagnosis.

Since such investigation has not been conducted on a large scale for the EU/EEA so far, we aimed to describe and compare TB patient characteristics of migrant subpopulations reported from low TB incidence European countries to the European Surveillance System (TESSy) to elucidate whether differences exist in the patient profiles within these subpopulations, and in comparison to the overall migrant TB patient population, and hence inform prevention and care strategies directed at those with highest TB risk.

## Methods

### Study design

This is a cross-sectional descriptive epidemiological study. We utilised the STROBE checklist for cross-sectional studies [9].

### Study population and setting

Data on migrants with TB provided by low TB incidence EU/EEA countries, Switzerland and the United Kingdom (UK) to The European Surveillance System (TESSy) were released by European Centre for Disease Prevention and Control (ECDC) for the study period 2014 to 2020. Data on TB patients were not available in TESSy from Switzerland for 2019–2020 and from the UK for 2020.

#### Definitions

We used the European Union Commission TB surveillance case definition [10]. No exclusions on case classifications or previous diagnosis were applied. Low TB incidence countries were defined as having a median TB crude incidence rate (CIR) of < 10 per 100,000 population for the study period [11]. Previous history of TB was defined as having a previous TB diagnosis reported to TESSy.

Resistance to any first line anti-TB drug was defined as resistance to one or more of the following drugs: isoniazid, rifampicin, ethambutol or pyrazinamide. Multi-drug resistance was defined as resistance to both isoniazid and rifampicin. Data were not available in TESSy to indicate whether drug-resistance was primary or acquired.

Migrants with TB were defined as TB patients reported with country of birth different to the destination country where TB was notified. Where country of birth was not available, country of nationality was used as the country of origin. Countries of origin were derived from the list of 245 countries and territories used by TESSy. No restrictions were applied on duration of residence in the destination country. No information was available on prior residence in another country other than their country of origin or the patient's legal status within the destination country.

#### Inclusion criteria

Patients were excluded from the analysis if neither country of birth nor nationality were reported (*n* = 8,047). Liechtenstein was excluded due to small sample size (*n* = 2). As a result, 21 EU/EEA countries (Austria, Belgium, Croatia, Cyprus, Czechia, Denmark, Finland, France, Germany, Greece, Hungary, Iceland, Ireland, Italy, Luxemburg, Netherlands, Norway, Slovakia, Slovenia, Spain, Sweden), Switzerland and the UK remained in the study. Drug-resistance surveillance data were incomplete for France, Italy, Spain and UK (data available for < 70% of patients). These countries were excluded from the analysis of resistance profiles.

### Statistical analysis

Counts and proportions of the following patient characteristics were calculated: age, sex, people living with HIV (PLWH, restricted to destination countries with ≥ 50% completeness), site of disease, previous TB diagnosis and drug-resistance profile. The full list of variables extracted from TESSy is included in the supplement (Table S1). Given that our data are derived from a registry sample without random selection, the notion of a target population becomes ambiguous, and therefore statistical tests/p-values are not presented as they would lack a meaningful context for generalization [12]. Data were received from TESSy as a Stata file and R software was used for the analysis [13].

#### Stratification of patient characteristics analysis

Patient characteristics of different migrant sub-populations were compared according to the three groups outlined below.

- Median incidence categories based on WHO estimates

The median TB incidence estimate per 100,000 population for each origin country for the study period was calculated using the annual estimates published by the WHO [14]. Origin countries were then classified as one of the following incidence categories: low = 0.0–9.9, medium = 10.0–39.9, high = 40.0–99.9 and very high ≥ 100. Incidence estimates were not available from WHO for 17 origin countries or territories within the study population (*n* = 219 patients, 0.2%).

b) Incidence categories based on origin country crude incidence rates (CIRs) estimated in the study population

Each origin country was also categorised as either ranking within the top 10 countries with highest TB CIRs or below, as derived from a partner publication using this study dataset [15]. Methods are described in full elsewhere but in brief: annual migrant TB CIRs per 100,000 population were calculated for 20 of the 23 destination countries^1^ in this study using Eurostat or national census population denominators stratified by country of birth. The ten origin countries or autonomous territories with the highest CIRs according to that study were Chad, Congo, Eritrea, Ethiopia, Gambia, Greenland (an autonomous territory within the Kingdom of Denmark), Guinea, Guinea-Bissau, Somalia and Sudan.

c) Categories based on interval between arrival and TB notification

The interval between arrival in the destination country and notification with TB was calculated as reporting year minus the year of arrival. We categorized the interval in four groups: < 2 years, 2–4 years, 5–9 years and ≥ 10 years. To minimise bias associated with high levels of missing data, analysis was restricted to destination countries which reported year of arrival for ≥ 70% of migrants with TB. As year of arrival was introduced for patients diagnosed from 2017 onwards, but no data was available for UK in 2020, analysis was further restricted to the reporting years 2017–2019. A sensitivity analysis for the period 2017–2020 is presented (Table S3).

### Protection of human subjects

As this was a secondary data analysis of anonymized data with no patient interaction or interventions, this did not meet the criteria of research using personally identifiable data which requires approval by a research ethics committee under the Helsinki Declaration [16]. All data processing was compliant with the General Data Protection Regulations [17].

## Results

### Patient characteristics – all migrant TB patients

A total of 114,370 migrants with TB from 221 origin countries were reported from 23 low TB incidence European countries in 2014–2020. Origin countries with the highest number of patients were India (*n* = 9,631), Romania (*n* = 8,558), and Pakistan (*n* = 7,718).

**Fig. 1:**
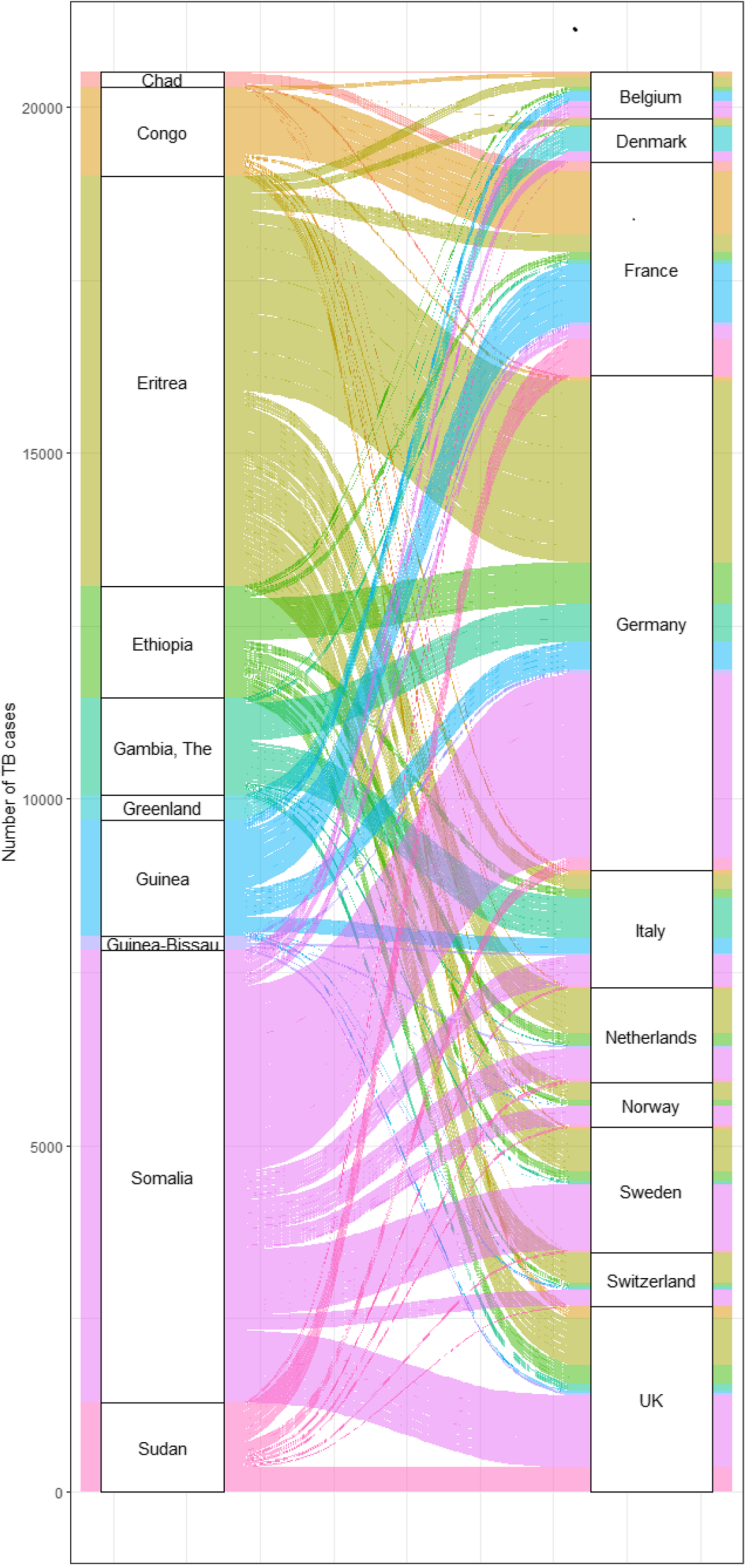
Alluvial plot for 10 countries of origin with highest TB CIRs among migrants and their 10 most common destination countries (2014–2020)

The destination and numbers of migrants with TB varied depending on their origin country. While patients from Romania were reported by all 23 destination countries, with the highest proportion reported by Italy (31.2%), most patients from Algeria were reported by France (77.2%, *n* = 1,470) with smaller numbers distributed among 15 other destination countries. A similar phenomenon was observed between the 10 origin countries with highest TB CIRs and their 10 most common destination countries (Fig. 1). Patients from Somalia (*n* = 7,141) were reported by 21 countries while patients from Chad (*n* = 209) were mostly reported by France (71.3%) with very small numbers reported by nine other destination countries.

In most countries, the highest proportion of migrants with TB originated from very high TB incidence countries (58.1%) and ranged from 3.1% in Croatia to 73.9% in the UK. In Hungary 63.7% of patients originated from high TB incidence countries whereas in Croatia 86.2% of patients came from medium incidence countries. The highest proportion of migrants with TB from low incidence countries was reported from Czechia (16.0%) (Supplementary Figure S1).

Patient characteristics are displayed according to the incidence category of the origin country (Table 1) and according to their ranking among top 10 origin countries with the highest TB CIRs among migrants (Table 2).

**Table 1:** Patient characteristics of migrants with TB in 23 low TB incidence European countries by WHO TB incidence of country of origin, 2014–2020 (*n* = 114,151)

Characteristic	Overall		Low (< 10/100,000)		Medium (10–39/100,000)		High (40–99/100,000)		Very high (≥ 100/100,000)	
	Number (Median)	Percentage/ratio	Number (Median)	Percentage/ratio	Number (Median)	Percentage/ratio	Number (Median)	Percentage/ratio	Number (Median)	Percentage/ratio
Total patients	***N*** = 114,151^b^		3,406	3.0%	14,289	12.5%	30,045	26.3%	66,411	58.2%
Median age (Mean age)	34 (38)		49 (48)		42 (44)		33 (36)		33 (36)	
*(Missing)*	*2,712*		*34*		*337*		*850*		*1,491*	
Sex										
* Male to Female ratio*		1.8		1.8		1.9	2.0			1.6
Diagnostic site										
* Pulmonary*	73,292	64.6%	2,550	75.3%	10,832	76.5%	22,587	75.6%	37,323	56.5%
* Extrapulmonary*	40,147	35.4%	838	24.7%	3,335	23.5%	7,291	24.4%	28,683	43.5%
* (Missing)*	*712*		*18*		*122*		*167*		*405*	
Previous TB diagnosis										
* Yes*	8,867	10.9%	245	9.0%	1,126	10.9%	2,808	13.7%	4,688	9.8%
* No*	72,503	89.1%	2,481	91.0%	9,186	89.1%	17,640	86.3%	43,196	90.2%
* (Missing)*	*32,781*		*680*		*3,977*		*9,597*		*18,527*	
People living with HIV^c^	***N*** = 14,091		***N*** = 565		***N*** = 2,039		***N*** = 3,478		***N*** = 8,009	
*Positive*	573	5.9%	15	4.6%	46	3.3%	140	5.6%	372	6.7%
*Negative*	9,156	94.1%	311	95.4%	1,346	96.7%	2,345	94.4%	5,154	93.3%
*(Missing)*	*4,362*		*239*		*647*		*993*		*2,483*	
Any first-line anti-TB drug resistance^d^	***N*** = 43,156		***N*** = 1,533		***N*** = 7,848		***N*** = 12,346		***N*** = 21,429	
*Yes*	4,241	9.8%	84	5.5%	591	7.5%	1,509	12.2%	2,057	9.6%
*No*	38,915	90.2%	1,449	94.5%	7,257	92.5%	10,837	87.8%	19,372	90.4%
MDR-TB^d^	***N*** = 43,156		***N*** = 1,533		***N*** = 7,848		***N*** = 12,346		***N*** = 21,429	
*Yes*	1,228	3.2%	19	1.4%	124	1.8%	658	6.0%	427	2.3%
*No*	36,702	96.8%	1,356	98.6%	6,648	98.2%	10,275	94.0%	18,423	97.7%
*(Missing)*	*5,226*		*158*		*1,076*		*1,413*		*2,579*	

^b^WHO incidence estimates were not available for 219 patients from 17 origin countries reported to TESSy

^c^HIV status was reported for ≥ 50% of migrants with TB from 10 destination countries: Belgium, Cyprus, Czechia, Greece, Ireland, Iceland, Netherlands, Norway, Slovenia and Slovakia

^d^First line drug resistance and MDR-TB results are derived from a sub-sample of 43,156 patients from 19 destination countries with 70% completeness of first line drug susceptibility results, and an origin country which could be mapped to WHO incidence estimates

**Table 2:** Patient characteristics of migrants with TB for origin countries^e^ with 10 highest CIRs (*n* = 114,370)

			**Top 10 highest CIRs origin country**			
**Characteristic**	**Overall**		**Yes**		**No**	
	Number (Mean)	Percentage/ratio	Number (Mean)	Percentage/ratio	Number (Mean)	Percentage/ratio
Total patients	***N*** = 114,370		21,747	19.0%	92,623	81.0%
Median age years (Mean)	34 (38)		25 (28)		37 (40)	
*(Missing)*	*2,713*		*314*		*2,399*	
Sex						
* Male to Female ratio*		1.8		2.6		1.6
Diagnostic site						
* Pulmonary*	73,446	64.6%	13,055	60.4%	60,391	65.6%
* Extrapulmonary*	40,212	35.4%	8,572	39.6%)	31,640	34.4%
* (Missing)*	*712*		*120*		*592*	
Previous TB diagnosis						
* Yes*	8,883	10.9%	1,300	8.3%	7,583	11.5%
* No*	72,649	89.1%	14,282	91.7%	58,367	88.5%
* (Missing)*	*32,838*		*6,165*		*26,673*	
People living with HIV^f^	***N*** = 14,139		***N*** = 2,905	20.5%	***N*** = 11,234	79.5%
* Positive*	574	5.9%	85	3.8%	489	6.5%
* Negative*	9,191	94.1%	2,147	96.2%	7,044	93.5%
* (Missing)*	*4,374*		*673*		*3,701*	
Any first-line drug resistance^g^	***N*** = 43,314		***N*** = 12,134	28.0%	***N*** = 31,180	72.0%
* Yes*	4,251	9.8%	1,214	10.0%	3,037	9.7%
* No*	39,063	90.2%	10,920	90.0%	28,143	90.3%
MDR-TB^g^	***N*** = 43,314					
* Yes*	1,235	3.2%	340	3.2%	895	3.3%
* No*	36,831	96.8%	10,402	96.8%	26,429	96.7%
* (Missing)*	*5,248*		*1,392*		*3,856*	

^e^Origin countries with top 10 highest CIRs are: Chad, Republic of Congo, Eritrea, Ethiopia, Gambia, Guinea, Guinea Bissau, Greenland (Autonomous territory of Denmark), Sudan and Somalia

^f^HIV status was reported for ≥ 50% of migrants with TB from 10 destination countries: Belgium, Cyprus, Czechia, Greece, Ireland, Iceland, Netherlands, Norway, Slovenia and Slovakia

^g^First line drug resistance and MDR-TB results are derived from a sub-sample of 43,314 patients from 19 destination countries with 70% completeness of first line drug susceptibility results

#### Age

The highest proportion of patients was observed in the 25–34 year age group (Supplementary Figure S2) with a median age of 34 years. A younger median age was observed in patients originating from the 10 origin countries with the highest CIRs (25 years) whereas higher median ages were observed among patients originating from low and medium incidence countries (49 and 42 years respectively).

#### Sex

The male to female ratio was 1.8 overall with a higher ratio observed among patients originating from the 10 origin countries with the highest CIRs (2.6).

#### People living with HIV

Information for HIV status was missing for 87.1% of our study population, with only 10 destination countries reporting HIV status for ≥ 50% of migrants with TB. Where HIV status was known, 5.9% of patients were PLWH. The highest proportion of PLWH was observed among migrant patients originating from very high incidence countries (6.7%).

#### Site of disease

The overall proportion of pulmonary TB (PTB) among migrant patients was 64.6% with the highest levels reported by Hungary (96.9%) and the lowest by the UK (49.4%). Patients originating from very high TB incidence countries had a lower proportion of PTB (56.5%) compared to patients from low, medium and high incidence countries (75.3%−76.5%). Similarly, the proportion of PTB was lower for the origin countries with 10 highest CIRs (60.4% vs 65.6%).

#### Previous TB diagnosis

Information on previous TB diagnosis was available for 71.3% of migrants with TB. Where reported, previously diagnosed patients accounted for 10.9% of all patients, with the highest levels reported by Slovakia (18.4%). The highest levels of previous TB diagnoses were observed among patients originating from high incidence countries (13.7%). Origin countries with the 10 highest CIRs had lower levels of previous diagnosis (8.3% vs 11.5%).

#### Drug resistance

First-line drug-resistance results were available for 43,314 patients (86.0%) from 19 destination countries. Of these patients, infection with a first-line drug resistant strain was reported in 9.8% and multi-drug resistant (MDR) strains in 3.2%.

The highest level of first-line drug-resistance was reported by Finland (12.8%) while the lowest level was reported by Croatia (1.3%). The highest level of MDR-TB was reported by Hungary (7.8%). Higher levels of infection with first-line drug resistant (12.2%) and MDR (6.0%) strains were observed in migrants with TB originating from high TB incidence countries.

Compared to patients without first-line drug-resistance, infection with a drug-resistant strain was found more often among patients with a previous TB diagnosis (14.4% vs. 8.8%) and among patients originating from high TB incidence countries (Table 3). Compared to patients without MDR-TB, patients infected with MDR-TB strains had a higher proportion diagnosed with PTB (83.0% vs 72.4%), previous TB diagnoses (28.7% vs 7.8%) and more often originated from high TB incidence countries (53.6% vs 28.0%).

**Table 3:** Patient characteristics of migrants with TB with drug-susceptibility data^h^ (*n* = 43,314)

Characteristic	First line anti-TB drug resistance						MDR-TB			
	**Overall**		**Yes**		**No**		**Yes**		**No**	
	Number (Mean)	Percentage/ratio	Number (Mean)	Percentage/ratio	Number (Mean)	Percentage/ratio	Number (Mean)	Percentage/ratio	Number (Mean)	Percentage/ratio
Total patients	N = 43,314		4,251	9.8%	39,063	90.2%	1,235	3.2%	36,831	69.8%
Median age (Mean age)	32 (37)		32 (35)		32 (37)		31 (33)		32 (37)	
*(Missing)*	*20*		*2*		*18*		*1*		*15*	
Sex										
* Male to Female ratio*	1.9		1.7		1.9		2.1		1.9	
Diagnostic site										
* Pulmonary*	31,166	72.1%	3,101	73.1%	28,065	72.0%	1,023	83.0%	26,619	72.4%
* Extrapulmonary*	12,051	27.9%	1,140	26.9%	10,911	28.0%	209	17.0%	10,168	27.6%
* (Missing)*	*97*		*10*		*87*		*3*		*44*	
Previous TB diagnosis										
* Yes*	3,214	9.3%	486	14.4%	2,728	8.8%	280	28.7%	2,298	7.8%
* No*	31,223	90.7%	2,888	85.6%	28,335	91.2%	696	71.3%	27,124	92.2%
* (Missing)*	8,877		877		8,000		259		7,409	
WHO incidence estimate category										
* Low*	1,533	3.6%	84	2.0%	1,449	3.7%	19	1.5%	1,356	3.7%
* Medium*	7,848	18.2%	591	13.9%	7,257	18.6%	124	10.1%	6,648	18.1%
* High*	12,346	28.6%	1,509	35.6%	10,837	27.8%	658	53.6%	10,275	28.0%
* Very high*	21,429	49.7%	2,057	48.5%	19,372	49.8%	427	34.8%	18,423	50.2%
* (Missing)*	*158*		*10*		*148*		*7*		*129*	

^h^First line drug resistance and MDR-TB results are derived from a sub-sample of 43,314 patients from 19 destination countries with 70% completeness of first line drug susceptibility results

#### Temporal trends

Patient characteristics examined remained relatively stable over time (Supplementary Table S2). The annual number of notifications by destination country is shown in Supplementary Table S4.

Patient characteristics by interval between arrival and notification with TB 2017–2019.

**Fig. 2:**
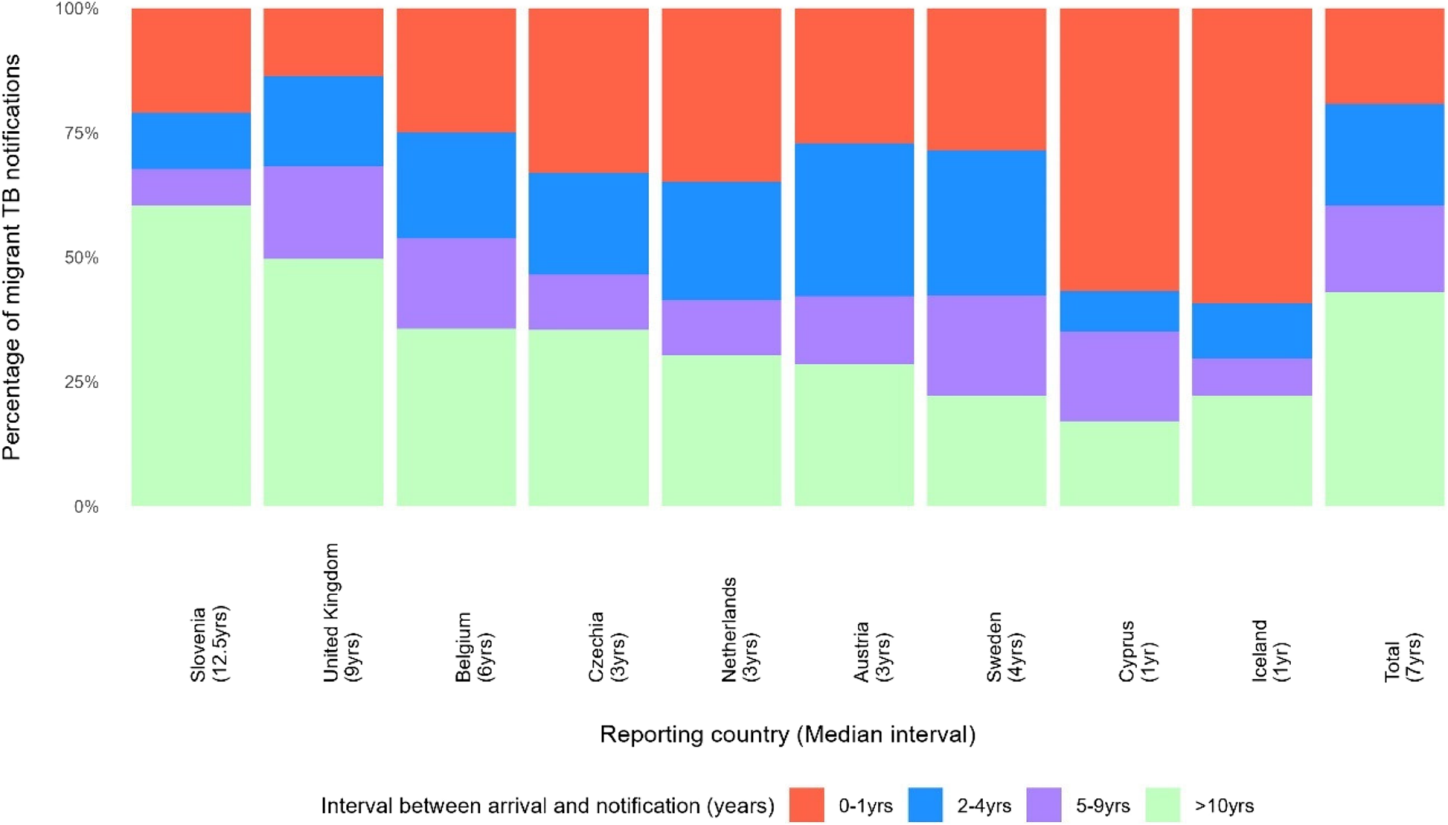
Interval between arrival in destination country and notification with TB by destination country; 2017–2019

Nine destination countries reported year of arrival for 15,646 migrants with TB (Fig. 2). The interval between arrival and notification ranged from 0–84 years (mean = 12.3 years; median = 7 years) and remained relatively stable over time. Of these, 19.2% of patients were reported as being notified with TB < 2 calendar years of arrival in the destination country, 20.4% within 2–4 years, 17.4% within 5–9 years and 43.1% 10 or more years after arrival. Time between arrival and notification with TB varied according to destination country with the longest median interval reported by Slovenia (12.5 years) and the UK (9 years) and the shortest by Cyprus and Iceland (1 year).

In contrast to migrants with TB residing longer in the destination country, migrant patients notified with TB < 2 years after arrival had higher proportions of patients diagnosed with PTB and MDR-TB as well as higher proportions of people living with HIV. Proportions of PTB and MDR-TB declined with increasing time after arrival. Younger median ages were observed among patients with TB notified < 2 years after arrival (28 years) and between 2–4 years (29 years) (Table 4).

**Table 4:** Patient characteristics of migrants with TB according to the interval between arrival in the destination country and notification with TB; 2017–2019 (*n* = 15,646)

Characteristic	Overall^i^		years		2–4 years		5–9 years		≥ 10 years	
	Number (Mean)	Percentage/ratio	Number (Mean)	Percentage/ratio	Number (Mean)	Percentage/ratio	Number (Mean)	Percentage/ratio	Number (Mean)	Percentage/ratio
	**15,646**		**2,999**	**19.2%**	**3,184**	**20.4%**	**2,723**	**17.4%**	**6,740**	**43.1%**
Median age (Mean age)	37 (40.4)		28 (30.6)		29 (31.4)		34 (36.9)		49 (50.5)	
*(Missing)*	*1*		*0*		*0*		*0*		*1*	
Sex										
* Male to Female ratio*	1.5		1.6		1.7		1.3		1.5	
WHO incidence estimate category										
* Low*	641	4.1%	80	2.7%	100	3.1%	87	3.2%	374	5.6%
* Medium*	1,581	10%	258	8.6%	296	9.3%	258	9.5%	769	11%
* High*	3,415	22%	1,056	35%	1,015	32%	583	21%	761	11%
* Very high*	9,997	64%	1,604	54%	1,772	56%	1,795	66%	4,826	72%
* (Missing)*	*12*		*1*		*1*		*0*		*10*	
Diagnostic site										
* Pulmonary*	8,811	56%	1,994	67%	1,807	57%	1,496	55%	3,514	52%
* Extrapulmonary*	6,824	44%	1,004	33%	1,376	43%	1,224	45%	3,220	48%
* (Missing)*	*11*		*1*		*1*		*3*		*6*	
Previous TB diagnosis										
* Yes*	991	6.7%	170	6.2%	171	5.7%	169	6.5%	481	7.4%
* No*	13,773	93%	2,560	94%	2,809	94%	2,423	93%	5,981	93%
* (Missing)*	*882*		*269*		*204*		*131*		*278*	
People living with HIV^j^	***N*** = 3,509		***N*** = 1,078		***N*** = 745		***N*** = 500		***N*** = 1,186	
* Positive*	138	5.6%	52	6.6%	24	4.6%	14	4.1%	48	6.0%
* Negative*	2,305	94%	732	93%	494	95%	331	96%	748	94%
* (Missing)*	*1,066*		*294*		*227*		*155*		*390*	
Any 1 st line drug resistance^k^	***N*** = 4,153		***N*** = 1,172		***N*** = 1,053		***N*** = 644		***N*** = 1,284	
* Yes*	473	11%	141	12%	135	13%	81	13%	116	9.0%
* No*	3,680	89%	1,031	88%	918	87%	563	87%	1,168	91%
MDR-TB	N = 4,153		N = 1,172		N = 1,053		N = 644		N = 1,284	
* Yes*	116	2.8%	51	4.4%	32	3.1%	16	2.5%	17	1.3%

^i^Destinations countries: Austria, Belgium, Cyprus, Czechia, Iceland, Netherlands, Sweden, Slovenia and the UK

^j^HIV status was reported for ≥ 50% of migrants with TB from six destination countries (Belgium, Cyprus, Czechia, Iceland, Netherlands and Slovenia) in addition to having ≥ 70% completeness for year of arrival

^k^First line drug resistance and MDR-TB results are derived from a sub-sample of 4,153 patients from 8 destination countries with 70% completeness of first line drug susceptibility results

Among migrants with TB from low and medium incidence origin countries, the proportions in each arrival interval remained relatively stable with the highest proportions observed in the ≥ 10 years interval (5.6% and 11%, respectively). For patients originating from high incidence countries the proportions in each arrival category declined with time after arrival (from 35% in the < 2 years interval to 11% in the ≥ 10 years interval). In contrast, among migrants with TB from very high incidence origin countries the proportion increased from 54 to 72%. While patient numbers in the ≥ 10 years arrival interval were comparable to combined numbers in the < 10 years categories for low (374 vs 267), medium (769 vs 812) and very high (4,826 vs 5,171) incidence countries, they were lower for high incidence countries (761 vs 2,654).

In Table 4, we presented column-wise percentage values for comparison between different origin country incidence categories. A complimentary view on those results is achieved by comparing the numbers in the rows, according to interval between arrival and notification. For low and medium incidence countries the proportion of patients diagnosed within the arrival intervals of 0–1, 2–4 and 5–9 years did not fluctuate very much and corresponded to approximately 40–50% of total TB patients. However, when comparing high and very high incidence countries, the difference is noticeable. For high incidence countries there is a high proportion of 30.9% of patients notified within a year after arrival which remains high for 2–4 yrs (29.7%), before declining substantially thereafter (17.1 and 22.3%). Whereas for very high incidence countries the proportion in the time intervals below 10 years stays relatively stable and low (range: 16%−18%) but remains high with 48.3% in the arrival interval ≥ 10 years.

## Discussion

This study describes 114,370 migrants with TB from 221 origin countries, reported by 23 low TB incidence destination countries in Europe from 2014–2020. Unique patterns in patient characteristics were observed which varied according to origin and destination countries. The majority of migrants with TB originated from very high TB incidence countries (58.1%), with the proportion ranging from 3.1—73.9% depending on the destination country. Migrants with TB originating from very high incidence countries had the highest proportion of PLWH (6.7%). This patient group also had the lowest proportion presenting with PTB (56.5%). Migrants with TB from high incidence countries had higher proportions of previous TB diagnosis (13.7%) as well as infection with first line drug-resistant and MDR-TB strains (12.2% and 6.0%, respectively). Patients coming from the 10 countries with the highest TB CIRs were on average nine years younger (median age 25 vs 34 years) and more often male (male to female ratio 2.6 vs. 1.8) compared to all migrants with TB. Almost 20% of patients with available information were notified with TB within one year, and 43% ≥ 10 years after arrival. In accordance with the `typical´ case characteristics of migrants with TB previously described based on surveillance data, the majority of patients in our study were young and predominantly male adults [1, 18, 19].

The overall proportion of PLWH was 5.9% and highest in patients from very high TB incidence countries. However, as data on HIV status was only provided for 13% of the study population, the interpretation is difficult given the results may also be influenced by testing policies such as targeted screening at time of entry for persons originating from countries with substantial TB/HIV coinfection prevalence. Recently, an overall higher HIV prevalence ratio among migrant than in native-born populations was shown, with highest rates for asylum seekers [20]. Therefore, HIV should always be integrated in TB care for migrant patients [21].

We observed higher proportions of extrapulmonary TB (EPTB) in patients from very high incidence countries compared to migrants with TB overall. EPTB is more common in certain regions (e.g. South-East-Asia and Sub-Saharan Africa) than European-born populations [22]. EPTB has been linked to host genetic pre-dispositions and areas with higher TB and HIV prevalence but may also be influenced by a higher prevalence of infection with *Mycobacterium bovis* as observed in certain countries [23, 24]. Consequently, proportions of EPTB in the destination country will be influenced by the origins of the migrant population. As screening measures focus on the detection of pulmonary TB, underdiagnosis of EPTB is likely and requires increased vigilance within screening algorithms as well as heightened awareness in the general healthcare systems [5, 22, 25, 26].

Previous TB diagnosis was reported in 10.9% of patients with highest levels among those from high incidence countries (13.7%). In this study, 40% of migrants from high incidence origin countries came from Europe (mainly from Romania, *n* = 8,558) and 39% from African countries (almost half from Eritrea, *n* = 6,006). In the very high incidence origin category most patients were born in African (52%) and Asian (46%) countries with none born in the European countries. We hypothesise that the higher proportion of previously diagnosed TB among migrant patients from high incidence countries may be influenced by easier access to diagnosis in high-income European countries rather than an actual difference in the proportion of patients with previous disease episodes.

Overall, we observed 9.8% first-line drug-resistance and 3.2% MDR-TB. Both proportions were highest in patients from high incidence countries, which might reflect a different accessibility or availability of anti-TB medication. Purposeful medical migration for TB treatment or re-treatment could also play a role, especially as the proportion of patients with MDR-TB was highest within a year of arrival [27]. Since a notable proportion of patients from high incidence countries who were diagnosed within a year of arrival were citizens of the Eastern European member states of the EU/EEA, many of which have high levels of MDR-TB, a hope for better medical or easier-to-reach TB care could have been one of the motivations for emigration for some of them [27]. The higher MDR-TB rates seen in recently migrated patients could also result from more recent infection post-exposure along migration pathways in settings with high MDR-TB prevalence, like crowded refugee detention camps in Libya [18, 28].

The highlighted differences in the extent and destinations of migration are most likely related to language, historical and political reasons (e.g. migrants from Algeria in France, from Slovakia in Czechia, from the autonomous territory of Greenland in Denmark), prevailing crises and national migration policies. Understanding of the current local migrant population origins and migration pathways, as well as of specific disease characteristics (such as drug resistance) is important to provide tailored TB prevention and care, especially as further migration changes are anticipated given environmental and political instability [1, 29, 30]. Such information can also benefit both countries of origin, and those along the main migration routes [31]. However, reducing morbidity and mortality in high and very high incidence countries by strengthening their healthcare systems is considered crucial to improve TB prevention and care and to reach global TB elimination goals [1, 31].

Only nine destination countries provided sufficient information on the year of arrival, covering approximately 14% of the study population. Recently arrived migrants with TB were younger, showed higher proportions of pulmonary and MDR-TB, and mainly originated from high or very high incidence countries. These findings were also mirrored in analyses performed with French and Irish data (Personal communication: Jean-Paul Guthmann, 2024; Sarah Jackson, 2024). The higher proportion of PTB in recently arrived migrants with TB is likely influenced by chest X-ray-based active screening measures for refugees and asylum seekers [5, 25, 26].

In low incidence countries, TB disease in migrant populations is considered to be mostly due to reactivation of TB infection acquired in the origin country or on the migration route [28, 30, 31]. The risk of developing TB remains elevated for several years after arrival [6, 32]. Though our observations support these findings, we consider proportions of migrants with TB being reported two or more years after arrival considerably high (ca. 80%). The relatively stable proportion of 40–50% of patients from low and medium incidence countries being diagnosed with TB within the first nine years after arrival is understandable considering that those groups rarely undergo entry or pre-entry screening [5, 33]. For high incidence origin countries, the high proportion of 30.6% of patients notified with TB in the first year after arrival may be explained by active screening measures, but it is important to notice that it remains almost equally high 2–4 yrs after arrival, before declining substantially thereafter. Whereas for very high incidence origin countries the proportion in the time intervals below 10 years stays relatively stable and low (16–18%) but rises to 48.3% for the ≥ 10 years after arrival interval. Besides emphasising the potential need to expand screening beyond TB disease to TB infection and provision of preventive therapy, these findings also point out the importance of continuous awareness for TB among healthcare providers taking care of migrant populations, including the availability of dedicated information on the elevated risk for TB (despite a negative entry screening) and access to TB care. Differing proportions in the interval between arrival and notification among destination countries may result from national screening strategies, such as pre-entry screening in UK reducing the chance of presenting with TB at time of entry [34]. This may also be partly related to the proportion of patients diagnosed within the first year of arrival increasing along the WHO incidence categories (low = 12%, medium = 16%, and high = 31%), except for very high incidence countries (16%) where the majority of the patients in UK comes from. According to whole genome sequencing studies, TB acquisition by transmission in the destination countries is less likely than reactivation [35]. The TB risk can also be influenced by travelling back and forth to the countries of origin, as well as by structural and social determinants of health present in the destination country [1, 36].

The main limitations we encountered were related to poor data completeness. Year of entry, HIV status (still not reported by 11 out of 30 EU/EEA countries in 2023 [37], and drug susceptibility (including pre-XDR and XDR TB) need to be reported more thoroughly. Legal barriers to reporting of HIV status of TB patients exist in many European countries. Future research and surveillance system evaluations should investigate the barriers and enablers to reporting HIV status of people with TB in line with WHO recommendations [21]. Countries should aim to submit high quality data to TESSy to enhance understanding of the epidemiology of TB among migrants to facilitating improvements in TB care strategies, which benefits all European countries. To ensure standardised and comparable surveillance, clear and universal definitions for classifying incidence categories and recent versus non-recent migration are required. Another limitation is the lack of publicly available population denominator data for the different migrant subpopulations in the study, which would help understand whether the observed demographic characteristics are specific to TB patients or rather represent the migrant populations´ characteristics. For example, we cannot determine whether the over-representation of males among the migrants with TB reflects the higher risk of TB among men than women, or results from an underlying migrant population cohort effect. Figure 1 illustrates how unevenly migrant populations from different origin countries were distributed among the destination countries, indicating a high level of migration pattern-related heterogeneity, which has not been addressed by this study. Since information on migration movements between European destination countries and back-and-forth migration from origin and destination country are not available, potential biases cannot be considered. As we focussed on migrant subpopulations and effects related to the arrival interval, we did not compare our findings with patient characteristics of the respective non-migrant population.

Our study allows for comparison of migrant patient sub-populations, adding upon previous publications on TB epidemiology in migrant populations within the EU/EEA, which have approached migrants as a homogenous group and employed different definitions for migrants within the same analysis [38]. Our approach is reproducible by using an official data source and transparently reported methods. As most authors are engaged in national TB surveillance, background knowledge on country-specific data notification and reporting particularities facilitated interpretation of the results. The observed period depicts the years before the high levels of international migration observed in 2015/2016 and the developments, thereafter, including the time before the COVID-19 pandemic and the full-scale war in Ukraine, which have since influenced TB epidemiology [2, 30, 39, 40]. Future studies will need to consider how the latter affected migration in general, as well as both health care seeking and provision during and after these crises. Our findings may also serve as a referral point for future evaluations of the impact of the wider roll out of shorter treatment regimens. This descriptive study is also paired with a partner study describing crude TB incidence rates in the migrant subpopulations within this study, to support tailored screening strategies for identified subpopulations, for instance by limiting active screening procedures to migrants who are most at risk [15].

## Conclusions

Understanding the dynamics and demography of migration and its impact on TB epidemiology is necessary in order to improve and better tailor TB prevention and care in those who benefit most, and to evaluate effects of ongoing or newly implemented public health interventions. Such understanding can be progressed using the methods presented here but requires timely and complete data for all relevant variables for all TB patients (including year of arrival, drug resistance and HIV status). Screening contributes to TB prevention and control in migrant populations with increased risk for (MDR-) TB but aims at the detection of pulmonary TB. Given higher levels of extrapulmonary TB in the migrant population we must also strive to expand opportunities for EPTB diagnosis and care, as well as provision of HIV diagnosis and care. Furthermore, the need for intensified awareness and tailored, group-oriented, person-centred information on TB and the persisting TB risk despite programmatic screening, as well as barrier-free access to TB prevention, diagnosis and care remains important for years after migrants´ arrival, particularly for persons originating from high and very high TB incidence settings. Finally, strengthening the healthcare systems in the countries with a high burden of (MDR-) TB is crucial to reduce morbidity in migrant populations.

## Supplementary Information

Supplementary Material 1.

## Data Availability

The authors do not have authorisation to share the data used in this study based on the conditions of data access. The raw data used in this study are available upon reasonable request to the European Centre for Disease Prevention and Control at: https://www.ecdc.europa.eu/en/publications-data/request-tessy-data-research.

## Abbreviations

CIR: Crude incidence rates
ECDC: European Centre for Disease Prevention and Control
EPTB: Extrapulmonary TB
EU/EEA: European Union (EU) and European Economic Area (EEA)
HIV: Human immunodeficiency virus
TB: Tuberculosis
TBI: TB infection
TESSy: The European Surveillance System
MDR-TB: Multi drug-resistant TB
PLWHIV: People living with HIV
PTB: Pulmonary TB
WHO: World Health Organization
XDR-TB: Extensively drug-resistant TB

## Footnotes

1 No population denominator data were available for Croatia, Cyprus and Greece.

## References

[1] Lonnroth K, Mor Z, Erkens C, Bruchfeld J, Nathavitharana RR, Van Der Werf MJ, et al. Tuberculosis in migrants in low-incidence countries: Epidemiology and intervention entry points. International Journal of Tuberculosis and Lung Disease. 2017;21:624–36. 10.5588/ijtld.16.0845 28482956

[2] European Centre for Disease Prevention and Control / World Health Organization. European Centre for Disease Prevention and Control/WHO Regional Office for Europe. Tuberculosis surveillance and monitoring in Europe 2024 – 2022 data. Stockholm; 2024.

[3] Peters L, Engelen P-J, Cassimon D, Explaining refugee flows. Understanding the,. European refugee crisis through a real options lens. PLoS ONE. 2015;2023(18):e0284390. 10.1371/journal.pone.0284390 PMC10118136 37079636

[4] International Organization for Migration. World Migration Report 2024. 2024.

[5] Kunst H, Burman M, Arnesen TM, Fiebig L, Hergens MP, Kalkouni O, et al. Tuberculosis and latent tuberculous infection screening of migrants in Europe: comparative analysis of policies, surveillance systems and results. Int J Tuberc Lung Dis. 2017;21:840–51. 28786791 10.5588/ijtld.17.0036

[6] Pareek M, Greenaway C, Noori T, Munoz J, Zenner D. The impact of migration on tuberculosis epidemiology and control in high-income countries: a review. BMC Med. 2016;14:48. 27004556 10.1186/s12916-016-0595-5 PMC4804514

[7] Seedat F, Hargreaves S, Nellums LB, Ouyang J, Brown M, Friedland JS. How effective are approaches to migrant screening for infectious diseases in Europe? A systematic review. Lancet Infect Dis. 2018;18:e259–71. 29778396 10.1016/S1473-3099(18)30117-8

[8] Castelli F, Sulis G. Migration and infectious diseases. Clin Microbiol Infect. 2017;23:283–9. 28336382 10.1016/j.cmi.2017.03.012

[9] Skrivankova VW, Richmond RC, Woolf BAR, Yarmolinsky J, Davies NM, Swanson SA, et al. Strengthening the Reporting of Observational Studies in Epidemiology Using Mendelian Randomization. JAMA. 2021;326:1614. 34698778 10.1001/jama.2021.18236

[10] European Commission. EU Tuberculosis case definitions. Commission Implementing Decision (EU) 2018/945. Official Journal of the European Union. 2018.

[11] World Health Organization. WHO global lists of high burden countries for tuberculosis, TB/HIV and multidrug / rifampicin-resistant TB (MDR / RR-TB), 2021–2025. 2021.

[12] Bangdiwala SI. When to p and when not to p. Neurogastroenterology & Motility. 2023;35. 10.1111/nmo.14672 37668305

[13] R Core Team RF for SC. R: A language and environment for statistical computing. 2021.

[14] World Health Organization. 2022 Global Tuberculosis Report WHO, TB burden estimates 2014–2020. 2023; https://www.who.int/teams/global-tuberculosis-programme/data#csv_files.

[15] Domaszewska T, Koch A, Jackson S, Arrazola de Oñate W, Guthmann J-P, Hauer B, et al. Tuberculosis rates in immigrants to low-incidence European countries: epidemiological differences and similarities. In-press. 2025. 10.2807/1560-7917.ES.2025.30.11.2400489 PMC11927070 40116030

[16] World Medical Association. Declaration of Helsinki. JAMA. 2013;310:2191. 24141714 10.1001/jama.2013.281053

[17] Regulation (EU) 2016/679 of the European Parliament and of the Council. 2016.

[18] Meaza A, Tola HH, Eshetu K, Mindaye T, Medhin G, Gumi B. Tuberculosis among refugees and migrant populations: Systematic review. PLoS ONE. 2022;17:e0268696. 35679258 10.1371/journal.pone.0268696 PMC9182295

[19] Odone A, Tillmann T, Sandgren A, Williams G, Rechel B, Ingleby D, et al. Tuberculosis among migrant populations in the European Union and the European Economic Area. Eur J Public Health. 2015;25:506–12. 25500265 10.1093/eurpub/cku208 PMC4440450

[20] Santoso D, Asfia SKBM, Mello MB, Baggaley RC, Johnson CC, Chow EPF, et al. HIV prevalence ratio of international migrants compared to their native-born counterparts: A systematic review and meta-analysis. EClinicalMedicine. 2022;53:101661. 36147629 10.1016/j.eclinm.2022.101661 PMC9486043

[21] World Health Organization. Integrating the prevention and control of noncommunicable diseases in HIV/AIDS, tuberculosis, and sexual and reproductive health programmes: implementation guidance. Geneva; 2023.

[22] Hayward SE, Rustage K, Nellums LB, van der Werf MJ, Noori T, Boccia D, et al. Extrapulmonary tuberculosis among migrants in Europe, 1995 to 2017. Clin Microbiol Infect. 2021;27:1347.e1-1347.e7. 33352301 10.1016/j.cmi.2020.12.006 PMC8437049

[23] Sotgiu G, Falzon D, Hollo V, Ködmön C, Lefebvre N, Dadu A, et al. Determinants of site of tuberculosis disease: An analysis of European surveillance data from 2003 to 2014. PLoS ONE. 2017;12:e0186499. 29155819 10.1371/journal.pone.0186499 PMC5695811

[24] Majoor CJ, Magis-Escurra C, van Ingen J, Boeree MJ, van Soolingen D. Epidemiology of Mycobacterium bovis Disease in Humans, the Netherlands, 1993–2007. Emerg Infect Dis. 2011;17:457–63. 21392437 10.3201/eid1703.101111 PMC3166011

[25] Menezes D, Zenner D, Aldridge R, Anderson SR, de Vries G, Erkens C, et al. Country differences and determinants of yield in programmatic migrant TB screening in four European countries. Int J Tuberc Lung Dis. 2022;26:942–8. 36163670 10.5588/ijtld.22.0186 PMC7615138

[26] de Vries G, Guthmann JP, Häcker B, Hauer B, Nordstrand K, Nowinski A, et al. TB among refugees from Ukraine in European countries. IJTLD OPEN. 2024;1:166–73. 38988409 10.5588/ijtldopen.24.0062 PMC11231824

[27] Eimer J, Patimeteeporn C, Jensenius M, Gkrania-Klotsas E, Duvignaud A, Barnett ED, et al. Multidrug-resistant tuberculosis imported into low-incidence countries-a GeoSentinel analysis, 2008–2020. J Travel Med. 2021;28. 10.1093/jtm/taab069 PMC9638878 33987682

[28] Walker TM, Merker M, Knoblauch AM, Helbling P, Schoch OD, van der Werf MJ, et al. A cluster of multidrug-resistant Mycobacterium tuberculosis among patients arriving in Europe from the Horn of Africa: a molecular epidemiological study. Lancet Infect Dis. 2018;18:431–40. 29326013 10.1016/S1473-3099(18)30004-5 PMC5864516

[29] Kharwadkar S, Attanayake V, Duncan J, Navaratne N, Benson J. The impact of climate change on the risk factors for tuberculosis: A systematic review. Environ Res. 2022;212:113436. 35550808 10.1016/j.envres.2022.113436

[30] Dohál M, Dvořáková V, Šperková M, Pinková M, Ghodousi A, Omrani M, et al. Tuberculosis in Ukrainian War Refugees and Migrants in the Czech Republic and Slovakia: A Molecular Epidemiological Study. J Epidemiol Glob Health. 2023;14:35–44. 38048026 10.1007/s44197-023-00166-5 PMC11043285

[31] Cookson ST, Maloney SA. Keeping up with a world in motion: Screening strategies for migrating populations. Clin Infect Dis. 2017;65:1410–1. 29017254 10.1093/cid/cix508 PMC11539171

[32] Langholz Kristensen K, Ravn P, Petersen JH, Hargreaves S, Nellums LB, Friedland JS, et al. Long-term risk of tuberculosis among migrants according to migrant status: A cohort study. Int J Epidemiol. 2020;49:776–85. 32380550 10.1093/ije/dyaa063

[33] Braga S, Vieira M, Barbosa P, Ramos JP, Duarte R. Tuberculosis screening in the European migrant population: a scoping review of current practices. Breathe. 2024;20:230357. 38746905 10.1183/20734735.0357-2023 PMC11091716

[34] Berrocal-Almanza LC, Harris R, Lalor MK, Muzyamba MC, Were J, O’Connell A-M, et al. Effectiveness of pre-entry active tuberculosis and post-entry latent tuberculosis screening in new entrants to the UK: a retrospective, population-based cohort study. Lancet Infect Dis. 2019;19:1191–201. 31471131 10.1016/S1473-3099(19)30260-9

[35] Barniol J, Niemann S, Louis VR, Brodhun B, Dreweck C, Richter E, et al. Transmission dynamics of pulmonary tuberculosis between autochthonous and immigrant sub-populations. BMC Infect Dis. 2009;9:197. 19961606 10.1186/1471-2334-9-197 PMC3224697

[36] Hayward S, Harding RM, McShane H, Tanner R. Factors influencing the higher incidence of tuberculosis among migrants and ethnic minorities in the UK. F1000Res. 2018;7. 10.12688/f1000research.14476.1 PMC6107974 30210785

[37] European Centre for Disease Prevention and Control / World Health Organization. Tuberculosis surveillance and monitoring in Europe 2025 - 2023 data. 2025.

[38] Vasiliu A, Köhler N, Altpeter E, Ægisdóttir TR, Amerali M, de Oñate WA, et al. Tuberculosis incidence in foreign-born people residing in European countries in 2020. Eurosurveillance. 2023;28:2300051. 37855907 10.2807/1560-7917.ES.2023.28.42.2300051 PMC10588305

[39] Guthmann J-P, Fraisse P, Bonnet I, Robert J. Active tuberculosis screening among the displaced population fleeing Ukraine, France, February to October 2022. Eurosurveillance. 2023;28. 10.2807/1560-7917.ES.2023.28.12.2300155 PMC10037662 36951786

[40] Hauer B, Kröger S, Haas W, Brodhun B. Tuberculosis in times of war and crisis: Epidemiological trends and characteristics of patients born in Ukraine, Germany, 2022. Eurosurveillance. 2023;28. 10.2807/1560-7917.ES.2023.28.24.2300284 PMC10318937 37318760

