# Supplementary material for: Differences found in patient characteristics of migrant tuberculosis sub-populations within low TB incidence European countries, 2014–2020
Source: BMC Infect Dis. 2025 Jun 2;25:787. doi: 10.1186/s12879-025-11085-0 (PMC12128271; doi:10.1186/s12879-025-11085-0)
Supplement: Supplementary file 1 — Supplementary Material 1. [file 12879_2025_11085_MOESM1_ESM.docx]

## **Supplementary materials**

#### **Table S1: List of variables extracted from the European Surveillance System (TESSy)**

| **Variable** | **Full name** | **Coded value list** |
| --- | --- | --- |
| Age | Age |  |
| BornReportingCountry | Born in the country of report | YesNoUnk: |
| CountryOfBirth | Country of birth of patient | 245 countries and territories |
| CountryOfNationality | Country of nationality of patient | 245 countries and territories |
| DateOfEntryToCountry | Date of entry to country |  |
| DateUsedForStatistics | Date used for statistics |  |
| DiagnosedAnteMortem | TB diagnosed ante-mortem | YesNoUnk: |
| EnrolledToTreatment | Enrolment to treatment | N = No, patient didn’t start the treatment NA = Not applicable UNK = Unknown Y = Yes, treatment have been started |
| Gender | Gender | F = Female M = Male O = Other (e.g., transsexual) Unk = Unknown |
| HIVStatus | HIV status | NEG = Negative POS = Positive UNK = Unknown |
| MajorSiteOfTB | Major site of the disease | BONEOTHER = Bone/joint other than spine CNSOTHER = Central nervous system other than meningitis DISSEM = Disseminated EXTRAPULMNOTSPEC = Extrapulmonary, exact site unk  GASTROINTEST = Peritoneal/digestive LYMPHEXTHOR = Lymphatic extrathoracic LYMPHINTHOR = Lymphatic intrathoracic MENING = Meningitis O = Other PLEURAL = Pleural PULMONARY = Pulmonary (lung parenchyma, tracheobronchial tree, larynx) SPINE = Spine Unk = Unknown UROGEN = Genito-urinary |
| NationalityReportingCountry | Citizen of the country of report | YesNoUnk: |
| Outcome12Months | Outcome at 12 months | COMPLETED = Treatment completed CURED = Cured  DEFAULTED = Defaulted or Lost to follow-up DIEDOTHER = Died because of other cause  DIEDTB = Died because of TB  DIEDUNK = Cause of death unknown FAILED = Treatment failed NA = Not Applicable STILLTREATMENT = Still on treatment  TRANSFERRED = Transferred  Unk = Unknown outcome |
| Outcome24Months | Outcome at 24 months | COMPLETED = Treatment completed CURED = Cured  DEFAULTED = Defaulted or Lost to follow-up DIEDOTHER = Died because of other cause  DIEDTB = Died because of TB  DIEDUNK = Cause of death unknown FAILED = Treatment failed NA = Not Applicable STILLTREATMENT = Still on treatment  TRANSFERRED = Transferred  Unk = Unknown outcome |
| Outcome36Months | Outcome at 36 months | COMPLETED = Treatment completed CURED = Cured  DEFAULTED = Defaulted or Lost to follow-up DIEDOTHER = Died because of other cause  DIEDTB = Died because of TB  DIEDUNK = Cause of death unknown FAILED = Treatment failed NA = Not Applicable TRANSFERRED = Transferred  Unk = Unknown outcome |
| PrevDiagnosis | Previous diagnosis | YesNoUnk: |
| PrevTreatment | Previous anti-TB drug treatment | YesNoNAUnk: |
| RecordId | Record id |  |
| ReportingCountry | Reporting country |  |
| ResultCulture | Result of the test for diagnosis | N = Negative for Mycobacterium tuberculosis complex P = Positive for Mycobacterium tuberculosis complex Unk = Unknown |
| ResultMicroscopy | Result of test for acid-fast bacilli (AFB) | N = Negative for acid-fast bacilli (AFB) P = Positive for acid-fast bacilli (AFB) Unk = Unknown |
| ResultOtherTest | Additional lab test results | HISTOL = Detection of granulomata at histo-pathology NONUCLA = No detection of nucleic acid  NUCLA = Detection of M.tuberculosis complex nucleic acid in any sample NUCLAHISTOL = Detection of both nucleic acid and granulomata Unk = Unknown |


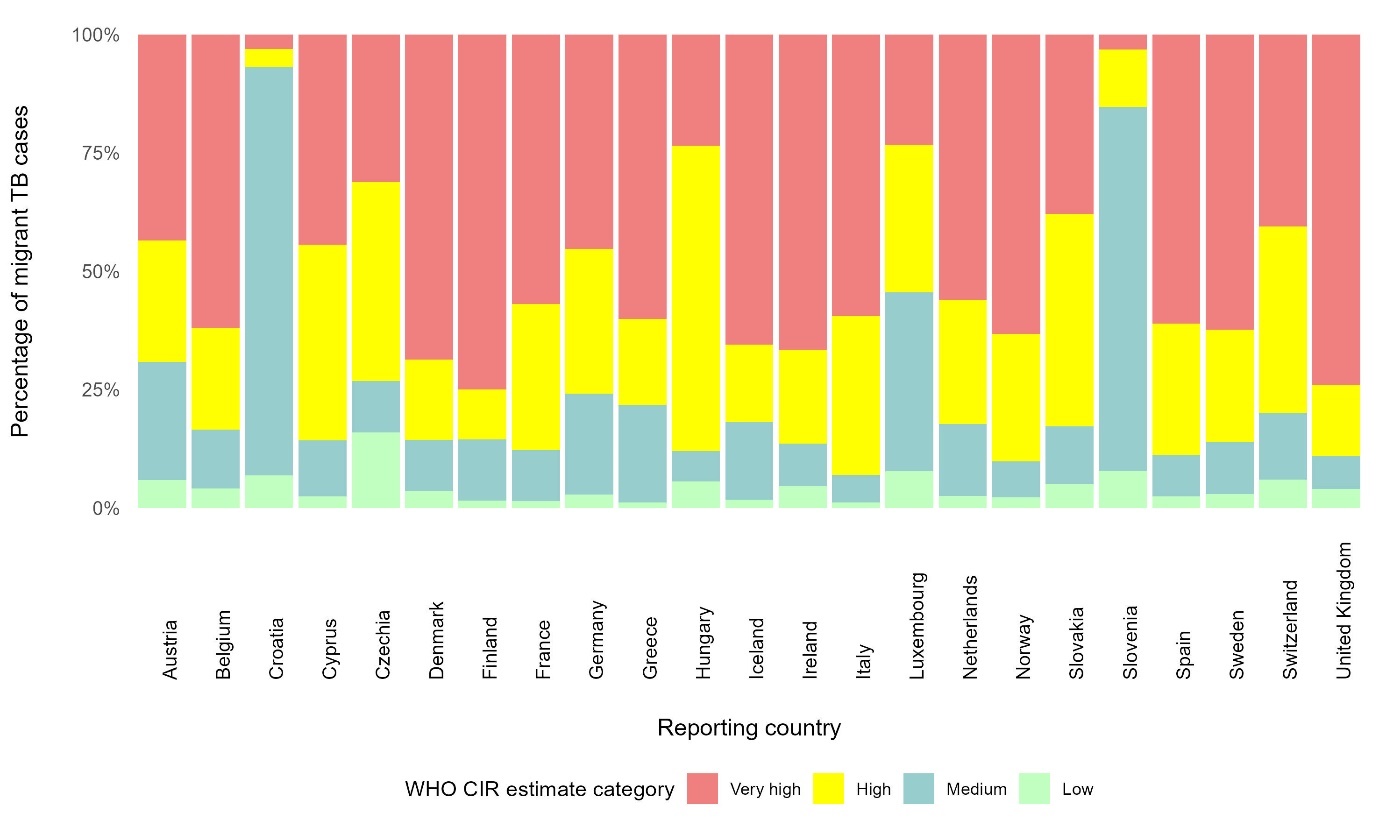


#### **Figure S1: Percentage of migrants with TB classified by reporting country and median WHO incidence estimate of the origin country**


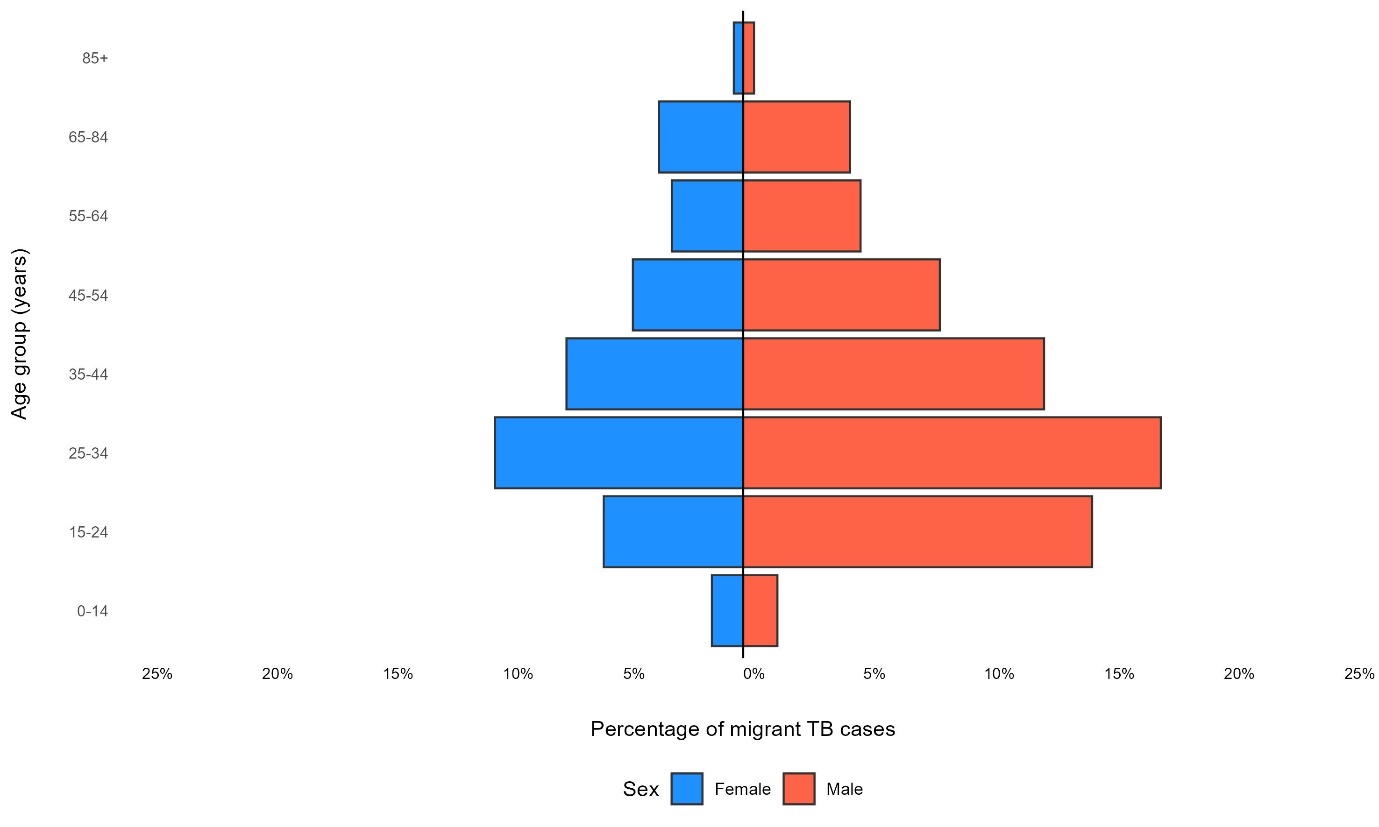


#### **Figure S1: Percentage of migrants with TB by age group and sex, 2014-2020**

#### **Table S2: Patient characteristics of migrants with TB by reporting year**

| **Characteristic** | **Overall**  N = 114,370 | **2014**  N = 14,061 | **2015**  N = 18,019 | **2016**  N = 18,769 | **2017**  N = 18,399 | **2018**  N = 17,829 | **2019**  N = 16,660 | **2020**  N = 10,633 |
| --- | --- | --- | --- | --- | --- | --- | --- | --- |
| **Median age**  **(Mean age)** | 34 (38) | 35 (38) | 34 (37) | 33 (37) | 34 (37) | 34 (38) | 35 (38) | 34 (38) |
| *(Missing)* | 2,713 | 17 | 2,626 | 38 | 10 | 13 | 3 | 6 |
| **Gender** |  |  |  |  |  |  |  |  |
| *Female* | 41,365 (36.3%) | 5,542 (39.5%) | 6,454 (36.0%) | 6,603 (35.4%) | 6,448 (35.1%) | 6,177 (34.7%) | 6,363 (38.2%) | 3,778 (35.6%) |
| *Male* | 72,730 (63.7%) | 8,505 (60.5%) | 11,460 (64.0%) | 12,069 (64.6%) | 11,924 (64.9%) | 11,634 (65.3%) | 10,289 (61.8%) | 6,849 (64.4%) |
| *(Missing)* | 275 | 14 | 105 | 97 | 27 | 18 | 8 | 6 |
| **People living with HIV** | |  |  |  |  |  |  |  |
| *Positive* | 992  (6.7%) | 143  (7.1%) | 153  (6.8%) | 131  (6.5%) | 18  (8.2%) | 93  (5.6%) | 181  (7.3%) | 108  (5.1%) |
| *Negative* | 13,746 (93.3%) | 1,864 (92.9%) | 2,089 (93.2%) | 1,879 (93.5%) | 2,061 (91.8%) | 1,569 (94.4%) | 2,289 (92.7%) | 1,995 (94.9%) |
| *(Missing)* | 99,632 | 12,054 | 15,777 | 16,759 | 16,155 | 16,167 | 14,190 | 8,530 |
| **Diagnostic site** |  |  |  |  |  |  |  |  |
| *Pulmonary* | 73,446 (64.6%) | 8,685 (61.9%) | 11,762 (65.5%) | 11,921 (64.7%) | 11,819 (64.4%) | 11,515 (64.9%) | 10,727 (64.8%) | 7,017 (66.3%) |
| *Extrapulmonary* | 40,212 (35.4%) | 5,338 (38.1%) | 6,207 (34.5%) | 6,507 (35.3%) | 6,520 (35.6%) | 6,234 (35.1%) | 5,838 (35.2%) | 3,568 (33.7%) |
| *(Missing)* | 712 | 38 | 50 | 341 | 60 | 80 | 95 | 48 |
| **Previous TB diagnosis** | |  |  |  |  |  |  |  |
| *Yes* | 8,883 (10.9%) | 1,103 (9.7%) | 1,399 (11.7%) | 1,801 (14.0%) | 1,593 (11.9%) | 1,205 (9.4%) | 1,111 (9.4%) | 671  (9.0%) |
| *No* | 72,649 (89.1%) | 10,219 (90.3%) | 10,604 (88.3%) | 11,063 (86.0%) | 11,764 (88.1%) | 11,584 (90.6%) | 10,659 (90.6%) | 6,756 (91.0%) |
| *(Missing)* | 32,838 | 2,739 | 6,016 | 5,905 | 5,042 | 5,040 | 4,890 | 3,206 |
| **Any 1st line drug resistance** | |  |  |  |  |  |  |  |
| *Yes* | 4,251 (9.8%) | 540  (9.0%) | 686  (9.7%) | 683  (9.3%) | 651 (10.0%) | 688 (10.8%) | 534  (9.8%) | 469 (10.2%) |
| *No* | 39,063 (90.2%) | 5,487 (91.0%) | 6,409 (90.3%) | 6,627 (90.7%) | 5,835 (90.0%) | 5,684 (89.2%) | 4,893 (90.2%) | 4,128 (89.8%) |
| **MDR-TB** |  |  |  |  |  |  |  |  |
| *Yes* | 1,235 (3.2%) | 176  (3.6%) | 212  (3.5%) | 196  (3.1%) | 201  (3.5%) | 185  (3.2%) | 144  (2.9%) | 121  (2.8%) |
| *No* | 36,831 (96.8%) | 4,697 (96.4%) | 5,846 (96.5%) | 6,048 (96.9%) | 5,620 (96.5%) | 5,626 (96.8%) | 4,834 (97.1%) | 4,160 (97.2%) |
| *(Missing)* | 5,248 | 1,154 | 1,037 | 1,066 | 665 | 561 | 449 | 316 |

#### **Table S3: Patient characteristics of migrants with TB according to the interval between arrival in the destination country^[[1]](#footnote-1)^ and notification with TB; 2017-2020 (9 destination countries, N = 17,021)**

| **Characteristic** | **Overall**,  N = 17,021 | **0-1**,  N = 3,365 | **2-4**,  N = 3,487 | **5-9**,  N = 2,983 | **10+**  N = 7,186 |
| --- | --- | --- | --- | --- | --- |
| **Median age (Mean age)** | 37.0 (40.2) | 28.0 (30.5) | 29.0 (31.4) | 34.0 (36.6) | 49.0 (50.5) |
| *(Missing)* | 1 | 0 | 0 | 0 | 1 |
| **Gender** |  |  |  |  |  |
| *F* | 6,783 (40%) | 1,270 (38%) | 1,332 (38%) | 1,283 (43%) | 2,898 (40%) |
| *M* | 10,237 (60%) | 2,094 (62%) | 2,155 (62%) | 1,700 (57%) | 4,288 (60%) |
| *(Missing)* | 1 | 1 | 0 | 0 | 0 |
| **WHO incidence estimate category** |  |  |  |  |  |
| *Low* | 690 (4.1%) | 88 (2.6%) | 111 (3.2%) | 89 (3.0%) | 402 (5.6%) |
| *Medium* | 1,779 (10%) | 296 (8.8%) | 318 (9.1%) | 287 (9.6%) | 878 (12%) |
| *High* | 3,759 (22%) | 1,168 (35%) | 1,107 (32%) | 653 (22%) | 831 (12%) |
| *Very high* | 10,779 (63%) | 1,812 (54%) | 1,950 (56%) | 1,954 (66%) | 5,063 (71%) |
| *(Missing)* | 14 | 1 | 1 | 0 | 12 |
| **People living with HIV^[[2]](#footnote-2)^** |  |  |  |  |  |
| *Positive* | 180 (5.8%) | 69 (7.1%) | 32 (4.8%) | 20 (4.4%) | 59 (5.8%) |
| *Negative* | 2,920 (94%) | 904 (93%) | 628 (95%) | 435 (96%) | 953 (94%) |
| *(Missing)* | 13,921 | 2,392 | 2,827 | 2,528 | 6,174 |
| **Diagnostic site** |  |  |  |  |  |
| *Extrapulmonary* | 7,328 (43%) | 1,121 (33%) | 1,507 (43%) | 1,327 (45%) | 3,373 (47%) |
| *Pulmonary* | 9,682 (57%) | 2,243 (67%) | 1,979 (57%) | 1,653 (55%) | 3,807 (53%) |
| *(Missing)* | 11 | 1 | 1 | 3 | 6 |
| **Previous TB diagnosis** |  |  |  |  |  |
| *Yes* | 1,080 (6.8%) | 194 (6.3%) | 198 (6.1%) | 182 (6.4%) | 506 (7.4%) |
| *No* | 14,911 (93%) | 2,863 (94%) | 3,044 (94%) | 2,645 (94%) | 6,359 (93%) |
| *(Missing)* | 1,030 | 308 | 245 | 156 | 321 |
| **Any 1st line drug resistance^[[3]](#footnote-3)^** | **N= 4,153** | **N = 1,172** | **N = 1,053** | **N = 644** | **N = 1,284** |
| *Yes* | 473 (11%) | 141 (12%) | 135 (13%) | 81 (13%) | 116 (9.0%) |
| *No* | 3,680 (89%) | 1,031 (88%) | 918 (87%) | 563 (87%) | 1,168 (91%) |
| **MDR-TB** |  |  |  |  |  |
| *Yes* | 116 (2.8%) | 51 (4.4%) | 32 (3.1%) | 16 (2.5%) | 17 (1.3%) |
| *No* | 3,978 (97%) | 1,101 (96%) | 1,009 (97%) | 622 (97%) | 1,246 (99%) |
| *(Missing)* | 59 | 20 | 12 | 6 | 21 |

#### **Table S4: Annual number and proportion of migrants with TB in low TB incidence European countries, 2014-2020**

| **Destination country** | **2014** | **2015** | **2016** | **2017** | **2018** | **2019** | **2020** | **Total** |
| --- | --- | --- | --- | --- | --- | --- | --- | --- |
| Austria | 378 (2.7%) | 402 (2.2%) | 432 (2.3%) | 400 (2.2%) | 324 (1.8%) | 275 (1.7%) | 247 (2.3%) | **2,458 (2.1%)** |
| Belgium | 611 (4.3%) | 641 (3.6%) | 698 (3.7%) | 629 (3.4%) | 661 (3.7%) | 676 (4.1%) | 593 (5.6%) | **4,509 (3.9%)** |
| Croatia | 64 (0.5%) | 79 (0.4%) | 1 (0.0%) | 4 (0.0%) | 4 (0.0%) | 6 (0.0%) | 2 (0.0%) | **160 (0.1%)** |
| Cyprus | 34 (0.2%) | 52 (0.3%) | 56 (0.3%) | 38 (0.2%) | 49 (0.3%) | 62 (0.4%) | 31 (0.3%) | **322 (0.3%)** |
| Czechia | 96 (0.7%) | 110 (0.6%) | 151 (0.8%) | 158 (0.9%) | 147 (0.8%) | 140 (0.8%) | 125 (1.2%) | **927 (0.8%)** |
| Denmark | 222 (1.6%) | 238 (1.3%) | 216 (1.2%) | 180 (1.0%) | 196 (1.1%) | 176 (1.1%) | 154 (1.4%) | **1,382 (1.2%)** |
| Finland | 86 (0.6%) | 116 (0.6%) | 109 (0.6%) | 93 (0.5%) | 92 (0.5%) | 94 (0.6%) | 63 (0.6%) | **653 (0.6%)** |
| France | 0 (0.0%) | 2,614 (14.5%) | 2,974 (15.8%) | 2,966 (16.1%) | 3,140 (17.6%) | 3,236 (19.4%) | 2,736 (25.7%) | **17,666 (15.4%)** |
| Germany | 2,740 (19.5%) | 4,083 (22.7%) | 4,319 (23.0%) | 3,932 (21.4%) | 3,995 (22.4%) | 3,413 (20.5%) | 2,895 (27.2%) | **25,377 (22.2%)** |
| Greece | 190 (1.4%) | 173 (1.0%) | 173 (0.9%) | 187 (1.0%) | 198 (1.1%) | 244 (1.5%) | 219 (2.1%) | **1,384 (1.2%)** |
| Hungary | 64 (0.5%) | 60 (0.3%) | 59 (0.3%) | 51 (0.3%) | 57 (0.3%) | 74 (0.4%) | 48 (0.5%) | **413 (0.4%)** |
| Iceland | 9 (0.1%) | 6 (0.0%) | 4 (0.0%) | 8 (0.0%) | 8 (0.0%) | 11 (0.1%) | 9 (0.1%) | **55 (0.0%)** |
| Ireland | 135 (1.0%) | 129 (0.7%) | 161 (0.9%) | 143 (0.8%) | 142 (0.8%) | 123 (0.7%) | 122 (1.1%) | **955 (0.8%)** |
| Italy | 1,775 (12.6%) | 1,713 (9.5%) | 1,919 (10.2%) | 2,294 (12.5%) | 2,241 (12.6%) | 1,871 (11.2%) | 1,239 (11.7%) | **13,052 (11.4%)** |
| Luxembourg | 20 (0.1%) | 19 (0.1%) | 22 (0.1%) | 25 (0.1%) | 34 (0.2%) | 45 (0.3%) | 28 (0.3%) | **193 (0.2%)** |
| Netherlands | 601 (4.3%) | 624 (3.5%) | 670 (3.6%) | 585 (3.2%) | 616 (3.5%) | 570 (3.4%) | 455 (4.3%) | **4,121 (3.6%)** |
| Norway | 302 (2.1%) | 277 (1.5%) | 262 (1.4%) | 231 (1.3%) | 179 (1.0%) | 147 (0.9%) | 130 (1.2%) | **1,528 (1.3%)** |
| Slovakia | 8 (0.1%) | 11 (0.1%) | 11 (0.1%) | 5 (0.0%) | 10 (0.1%) | 7 (0.0%) | 6 (0.1%) | **58 (0.1%)** |
| Slovenia | 36 (0.3%) | 50 (0.3%) | 43 (0.2%) | 45 (0.2%) | 44 (0.2%) | 36 (0.2%) | 26 (0.2%) | **280 (0.2%)** |
| Spain | 885 (6.3%) | 1,141 (6.3%) | 1,020 (5.4%) | 1,754 (9.5%) | 1,394 (7.8%) | 1,457 (8.7%) | 1,223 (11.5%) | **8,874 (7.8%)** |
| Sweden | 604 (4.3%) | 729 (4.0%) | 641 (3.4%) | 467 (2.5%) | 421 (2.4%) | 415 (2.5%) | 282 (2.7%) | **3,559 (3.1%)** |
| Switzerland | 357 (2.5%) | 417 (2.3%) | 481 (2.6%) | 408 (2.2%) | 372 (2.1%) | n/a | n/a | **2,035 (1.8%)** |
| United Kingdom | 4,844 (34.4%) | 4,335 (24.1%) | 4,347 (23.2%) | 3,796 (20.6%) | 3,505 (19.7%) | 3,582 (21.5%) | n/a | **24,409 (21.3%)** |
| **Total** | **14,061 (100.0%)** | **18,019 (100.0%)** | **18,769 (100.0%)** | **18,399 (100.0%)** | **17,829 (100.0%)** | **16,660 (100.0%)** | **10,633 (100.0%)** | **114,370 (100.0%)** |

n/a = no longer reporting to TESSy

1. Destinations countries: Austria, Belgium, Cyprus, Czechia, Iceland, Netherlands, Sweden, Slovenia and the UK. [↑](#footnote-ref-1)
2. HIV status was reported for ≥50% of migrants with TB by six destination countries (Belgium, Cyprus, Czechia, Iceland, Netherlands and Slovenia) in addition to having ≥70% completeness for year of arrival. [↑](#footnote-ref-2)
3. First line drug resistance and MDR-TB results are derived from a sub-sample of 4,153 patients from 8 destination countries with 70% completeness of first line drug susceptibility results. [↑](#footnote-ref-3)
